# Clinical implications of free triiodothyronine levels and diagnostic revisions in antibody-negative autoimmune encephalitis

**DOI:** 10.3389/fimmu.2026.1847846

**Published:** 2026-07-07

**Authors:** Ting Fang, Xinjie He, Junling Chen, Linhuan Huang, Yingyu Xie, Danni Li, Yinting Huang, Qi Lin, Houshi Zhou

**Affiliations:** 1 Department of Neurology, Shantou Central Hospital, Shantou, China; 2 Department of Cardiology, Shantou Central Hospital, Shantou, China

**Keywords:** antibody-negative autoimmune encephalitis, autoimmune encephalitis, low triiodothyronine syndrome, prognosis, revised diagnoses

## Abstract

**Background and objective:**

Low triiodothyronine (T3) syndrome has been associated with initial clinical severity and poor long-term functional outcomes in autoimmune encephalitis (AE). However, its prognostic implications in antibody-negative AE remain unclear. The objective of this study was to explore the clinical significance of low T3 syndrome in patients with antibody-negative AE, and to assess the clinical characteristics of patients reclassified with alternative diagnoses during follow-up.

**Methods:**

We conducted a retrospective cohort study of patients initially diagnosed with antibody-negative AE between January 2016 and June 2024. Patients were divided into two groups based on the presence or absence of low T3 syndrome. Demographics, clinical features, and ancillary test results were compared between the subgroups. Modified Rankin Scale (mRS) scores were used to evaluate neurological function during hospitalization and at the 12-month follow-up. In addition, we analyzed reclassified patients and their clinical outcomes to improve clinical management of antibody-negative AE.

**Results:**

Of the 84 patients initially diagnosed with antibody-negative AE at discharge, eight were reclassified during their disease course (non-autoimmune encephalitis, n=4; paraneoplastic encephalitis, n=3; neuropsychiatric systemic lupus erythematosus, n=1; these eight patients were defined as reclassified group) and were excluded from the antibody-negative AE group. A total of 76 patients were ultimately enrolled. Of these, 23.68% presented with low T3 syndrome during the acute phase. Subgroup analysis further showed that patients with low T3 syndrome had a higher incidence of consciousness disturbances (p = 0.048), more frequent motor impairments, and higher scores on the modified Rankin Scale (mRS) throughout hospitalization. Overall, 61.84% (47/76) of patients achieved a favorable prognosis, whereas 38.16% (29/76) had an unfavorable outcome. Notably, low T3 syndrome was associated with poor prognosis in univariable analysis but not after multivariable adjustment. Discharge mRS was an independent predictor of unfavorable outcome (OR 0.293, 95% CI 0.103-0.834, p = 0.021). Furthermore, the reclassified group demonstrated a higher recurrence rate compared with the antibody-negative AE group.

**Conclusion:**

Acute-phase low T3 syndrome is common in antibody-negative AE but appears to reflect disease severity rather than serving as an independent prognostic biomarker; discharge mRS is a more reliable predictor. Larger prospective studies are needed to clarify the prognostic role of thyroid hormone alterations in this population. Furthermore, a subset of patients (8/84, 9.5%) initially diagnosed with antibody-negative AE received alternative diagnoses after 12 months of follow-up, highlighting diagnostic uncertainty and the urgent need to identify clinical red flags for re-evaluation.

## Introduction

Autoimmune encephalitis (AE) is a severe immune-mediated inflammatory disorder of the central nervous system, characterized by diverse clinical manifestations including subacute or acute cognitive dysfunction, neuropsychiatric symptoms, new-onset seizures, movement disorders, and central hypoventilation. In recent years, the discovery of novel antibody biomarkers has significantly advanced research on autoimmune encephalitis (1–3). However, a substantial proportion of patients presenting with clinical features suggestive of AE test negative for known antibodies and are defined as having antibody-negative AE according to diagnostic criteria established in 2016 (4, 5). The diagnosis of this entity remains a significant clinical challenge due to the absence of a definitive serological biomarker and its heterogeneous, often non-specific presentation that overlaps with other conditions such as viral encephalitis, neurodegenerative diseases, and psychiatric disorders (6–8). Consequently, misdiagnosis is frequent, leading to delays in initiating appropriate immunotherapy, which is critical for improving outcomes.

Moreover, the prognostic stratification of antibody-negative AE is equally uncertain. Current markers of disease severity and outcome prediction are limited, primarily relying on clinical assessment, neuroimaging findings, and cerebrospinal fluid analysis (9). There is a pressing need to identify accessible and reliable biological markers that can reflect disease severity and predict recovery.

Low triiodothyronine (T3) syndrome is characterized by decreased free triiodothyronine (fT3) levels with normal or low thyroid-stimulating hormone (TSH) levels. fT3 plays a crucial role in neuroprotection and modulation of inflammation, and evidence has shown that low fT3 levels are associated with poorer outcomes in conditions such as stroke, traumatic brain injury, and intensive care unit (ICU) patients requiring invasive mechanical ventilation (10–12). In recent years, this syndrome has gained increasing recognition in neuroinflammatory disorders such as AE and neuromyelitis optica spectrum disorder (NMOSD), serving as a useful indicator of disease severity and disability (13–15). However, the role of low T3 syndrome in antibody-negative AE remains unclear.

To address this, our study investigated the correlation between low T3 syndrome and clinical manifestations as well as long-term neurological outcomes. Furthermore, we summarized the clinical features and outcomes of reclassified patients, aiming to enhance diagnostic accuracy and refine prognostic stratification for identify factors that could inform clinical management.

## Methods

### Study design

This study was conducted in accordance with the principles of the Declaration of Helsinki, and the protocol was approved by the Medical Ethics Committee of Shantou Central Hospital, Shantou, Guangdong, China (ethics number: [2025]-066).

We analyzed data from 84 Chinese Han patients initially suspected of having antibody-negative AE between January 2016 and June 2024 at Shantou Central Hospital, Shantou, Guangdong, China. Patient clinical information was obtained from medical records, outpatient visits, and telephone interviews.

Inclusion Criteria: (1) Patients aged ≥14 years; (2) fulfill the diagnostic criteria for possible AE (1): subacute onset of abnormalities in memory, mental status, or psychiatric symptoms, after reasonable exclusion of other potential causes, accompanied by at least one of the following new focal central nervous system (CNS) findings: unexplained seizures, cerebrospinal fluid (CSF) pleocytosis, or magnetic resonance imaging (MRI) features suggestive of encephalitis; (3) fulfilled the diagnostic criteria for antibody-negative but possible AE: rapid progression of abnormalities in memory, mental status, or psychiatric symptoms that cannot be attributed to a definite AE syndrome, with no characteristic auto-antibodies detected in either serum or CSF, and presence of at least two of the following indicators: MRI abnormalities suggestive of AE, AE-related CSF changes (such as pleocytosis, CSF-specific oligoclonal bands, or elevated IgG index), or brain biopsy showing inflammatory infiltrates, with other potential causes reasonably excluded.

Exclusion Criteria: (1) Infectious encephalitis confirmed by laboratory diagnosis of central nervous system infection, including infectious etiologies such as herpesviridae, respiratory viruses, enteroviruses, measles virus, Japanese B virus, John Cunningham (JC) virus, mycobacteria, fungi, Toxoplasma, and Cryptococcus; (2) patients with a confirmed diagnosis of toxic encephalopathy, metabolic encephalopathy, brain tumor, vitamin deficiency, alcohol-related encephalopathy, systemic autoimmune disease, epilepsy, or other neurological diseases prior to disease onset; (3) primary psychiatric disorders; (4) a history of intrinsic thyroid disease (hypothyroidism, hyperthyroidism, thyroiditis, or central hypothyroidism); presence of comorbidities that may lead to low T3 syndrome, including trauma, severe heart failure, chronic renal failure, acute myocardial infarction, severe liver disease; receiving antithyroid drug therapy, thyroid hormone replacement therapy, or using medications known to affect thyroid hormone secretion and metabolism. All enrolled patients were evaluated by two experienced neurologists according to the above criteria and procedures before inclusion.

### Laboratory testing

Blood samples for thyroid function assessment were collected from all patients within 24 hours of admission, prior to the initiation of corticosteroids or any other immunotherapy. Serum concentrations of thyroid hormones were quantified using a chemiluminescence immunoassay analyzer (Beckman). Normal reference ranges were as follows: fT3: 3.8–6.0 pmol/L; fT4: 7.9–14.4 pmol/L; TSH: 0.38–5.33 μIU/mL. Measurements were performed using a Siemens ADVIA Centaur XP automatic chemiluminescence immunoassay system. Low T3 syndrome was diagnosed according to the following criteria: a decreased serum fT3 level, in conjunction with low or normal levels of serum free thyroxine (fT4) and TSH.

### Clinical data collection

Clinical data of the enrolled patients, including age at onset, gender, clinical manifestations, prodromal infection, treatments, outcomes, follow-up status, and results of auxiliary examinations (including CSF analysis, serum analysis, electroencephalogram [EEG], and MRI), were reviewed. Early and timely treatment was defined as initiation of immunotherapy within 30 days after diagnosis of antibody-negative AE. Neurological impairment was assessed using the modified Rankin Scale (mRS) at admission, at peak disease severity, at discharge, and at 12 months after discharge. Based on mRS scores at 12 months post-discharge, patients were categorized into two groups: a favorable outcome group (mRS score ≤ 2) and a poor outcome group (mRS score > 2).

### Antibody testing

CSF and serum samples from all patients were screened prior to administering immunotherapy. Serum and/or CSF samples were tested using cell-based assays (CBAs) or immunodot assays for autoimmune encephalitis antibodies, including AQP-4, GFAP, MOG, NMDAR, AMPAR, GABAAR, GABABR, LGI1, CASPR2, IgLON5, DPPX, mGluR1, mGluR5, GlyR1, D2R, neurexin-3α, and paraneoplastic antibodies, including Hu, Yo, Ri, Ma1, Ma2, CV2, SOX1, Tr/DNER, Zic4, titin, PKC-γ, recoverin, GAD65, and amphiphysin. Antibody negativity was defined as the absence of autoimmune antibodies in both serum and CSF tests.

### Statistical analysis

Statistical analyses were performed using SPSS version 20.0 (SPSS, Chicago, IL, USA). Normally distributed continuous variables are expressed as mean ± standard deviation. Non-normally distributed data are presented as median and interquartile range (IQR). Categorical variables are expressed as percentages. The chi-square test or Fisher’s exact test was used for categorical variables, and the Mann–Whitney U test for continuous variables. Spearman’s correlation coefficient and Point-biserial correlation were used to evaluate correlations between low T3 syndrome and clinical characteristics as well as prognosis. Finally, univariate analysis was first performed with a significance level of p < 0.1 to identify factors related to long-term outcomes. Then multivariate logistic stepwise regression analyses were performed to validate and identify risk factors associated with these outcomes. The primary outcome was dichotomized into unfavorable outcome (coded as 1) and favorable outcome (coded as 0). p < 0.05 was defined as statistically significant.

## Results

### Baseline demographics and clinical features of patients with antibody-negative AE

Of the 84 patients initially diagnosed with antibody-negative AE at discharge, eight were reclassified during their disease course and were excluded from antibody-negative AE group. A total of 76 patients were ultimately enrolled. The patients’ demographic and clinical presentation are summarized in **Table 1**. Among the 76 antibody-negative AE patients, the median age at onset was 44 years (IQR: 14–85), with 22 females and 54 males, including 18 patients over 60 years of age. The median hospital stay was 13 days. The most frequently reported symptom was seizures (35/76, 46.05%), followed by headache (26/76, 34.21%), fever (22/76, 28.95%), movement disorders (20/76, 26.32%), and psychiatric symptoms (18/76, 23.68%). Other manifestations included altered consciousness, refractory status epilepticus (RSE), autonomic dysfunction, central hypoventilation, and bladder dysfunction. Additionally, 10 patients (13.16%) reported prodromal symptoms.

**Table 1:** Demographics, clinical profiles, treatment,and outcomes in patients with antibody-negative autoimmune encephalitis and subgroup comparisons.

Variables	Total (n =76)	Low T3 syndrome subgroup (n =18 )	Non-low T3 syndrome subgroup (n =58)	P value
Demographics				
Age at onset, (median, IQR)	44(14-85)	52(21-85)	43(14-81)	0.248
Age of onset ≥60	18(23.68)	4 (22.22)	14(24.14)	1
Female sex ,n(%)	22(28.95)	4(22.22)	18(31.03)	0.563
Clinical profiles				
The mRS score at admission, mean (SD)	2.96±1.03	3.33±0.69	2.84±1.09	0.077
Peak mRS scores, mean (SD)	3.0±0.99	3.44±0.62	2.86±1.05	0.005 *
mRS at discharge, mean (SD)	2.30±1.03	2.67±1.03	2.19±1.02	0.087
Hospital stay, median (IQR), days	13(3-84)	15(3-39)	12(3-84)	0.529
ICU admission,n(%)	5(6.58)	3(16.67)	2(3.45)	0.083
Comorbidities, n (%)				
Hypertension	13(17.11)	3(3.95)	10(17.24)	1
Diabetes	8(10.53)	1(5.56)	7(12.07)	0.729
Tumor	2(2.63)	0	2(3.45)	1
Elevated tumor markers	3(3.95)	1(5.56)	2(3.45)	1
Other concomitant non-neural autoantibodies in the serum	9(11.84)	2(11.11)	7(12.07)	1
Prodromal symptoms	10(13.16)	3(16.67)	7(12.07)	0.916
Clinical syndrome,n(%)				
Fever	22(28.95)	8(44.44)	14(24.14)	0.106
Headache	26(34.21)	3(16.67)	23(39.66)	0.131
Psychiatric symptoms	18(23.68)	4(22.22)	14(24.14)	1
Seizure	35(46.05)	9(50.00)	26(44.83)	0.701
RSE	8(10.53)	4(22.22)	4(6.90)	0.158
Movement disorders	20(26.32)	8(10.53)	12(20.69)	0.046 *
Autonomic dysfunction	6(7.89)	3(3.95)	3(5.17)	0.28
Altered consciousness	9(11.84)	5(27.78)	4(6.90)	0.048 *
Bladder dysfunction	4(5.26)	2(11.11)	2(3.45)	0.504
Central hypoventilation	6(7.89)	3(16.67)	3(5.17)	0.28
Blood tests				
WBC count, median (IQR), n× 109 /L	8.34(2.79-17.1)	7.92(2.79-17.02)	8.40(4.33-17.1)	0.217
CRP, median (IQR), mg/L	3.41(0.5-132)	3.71(0.5-56.5)	3.41(0.5-161)	0.769
D-Dimer,median (IQR), ug/L	465(50-4950)	745(90-7210)	385(50-4780)	0.107
Thyroid status				
fT3, median (IQR), pmol/L	4.42(0.92-11.91)	3.27(0.92-3.74)	4.66(3.81-11.91)	<0.01 *
fT4, median (IQR), pmol/L	11.6(7.39-17.76)	11.5(8.76-14.09)	11.8(7.39-17.76)	0.16
TSH, median (IQR), uIU/ml	1.15(0.11-4.57)	1.01(0.22-4.57)	1.28(0.11-4.72)	0.067
CSF findings, n(%)				
Increased intracranial pressure (cmH2O)	18(23.68)	3(16.67)	15(25.86)	0.628
Elevated white cell count (>5/μL)	23(30.26)	7(38.89)	16(27.59)	0.362
Elevated white cell count (> 20 white cell count/μl)	18(23.68)	7(38.89)	11(18.97)	0.082
Elevated protein (>45 mg/dL)	37(48.68)	9(50.00)	28(48.28)	0.898
MRI finding, n(%)				
Abnormal T2WI/FLAIR hyperintensities	59(77.63)	17(94.44)	42(72.41)	0.102
Cortex	32(42.11)	10(55.56)	22(37.93)	0.186
White matter	14(18.42)	5(27.78)	9(15.52)	0.241
Basal ganglia	11(14.47)	3(16.67)	8(13.80)	1
Thalamus	6(7.89)	2(11.11)	4(6.90)	0.937
Corpus callosum	5(6.58)	1(5.56)	4(6.90)	1
Hippocampus	21(27.63)	2(11.11)	19(32.76)	0.136
Brainstem	10(13.16)	4(22.22)	6(10.34)	0.366
Cerebellum	10(13.16)	2(11.11)	8(13.79)	1
infratentorial involvement EEG abnormalities,n(%)	16(21.05) 24(31.58)	6(33.33) 9(50.00)	10(17.24) 15(25.86)	0.143 0.054
Treatment, n(%)				
First-line immunotherapy				
Steroids	69(90.79)	18(100.00)	51(87.93)	0.28
IVIg	16(21.05)	6(33.33)	10(17.24)	0.143
Combined immunotherapy	16(21.05)	6(33.33)	10(17.24)	0.143
Second-line immunotherapy				
Delay of immunotherapy for ≥1 month	23(30.26)	7(38.89)	16(27.59)	0.362
Long-course immunotherapy	4(5.26)	2(11.11)	2(3.45)	0.504
Follow‐up outcomes, n(%)				
Good outcome (mRS<3)	47(61.84)	5(27.78)	42(72.41)	
Poor outcome (mRS 3–6)	29(38.16)	13(72.22)	16(27.59)	<0.001 *
mRS at 12 months after discharge, mean (SD)	2.16±1.46	3.0±1.64	1.90±1.31	0.004 *
Relapse	26(38.16)	9(50.00)	17(29.31)	0.106
Death	4(5.26)	2(11.11)	2(3.45)	0.504

IQR, interquartile range; SD, standard deviation; RSE, Refractory status epilepticus; MRI, magnetic resonance imaging; WBC, white blood cell; CRP, c-reactive protein; CSF, cerebrospinal fluid; fT3, free triiodothyronine; fT4, free thyroxine; TSH, thyroid stimulating hormone; ICU, intense care unit; mRS, modified Rankin scale; IVIg, intravenous immunoglobulin. *P<0.05.

**Figure 1:**
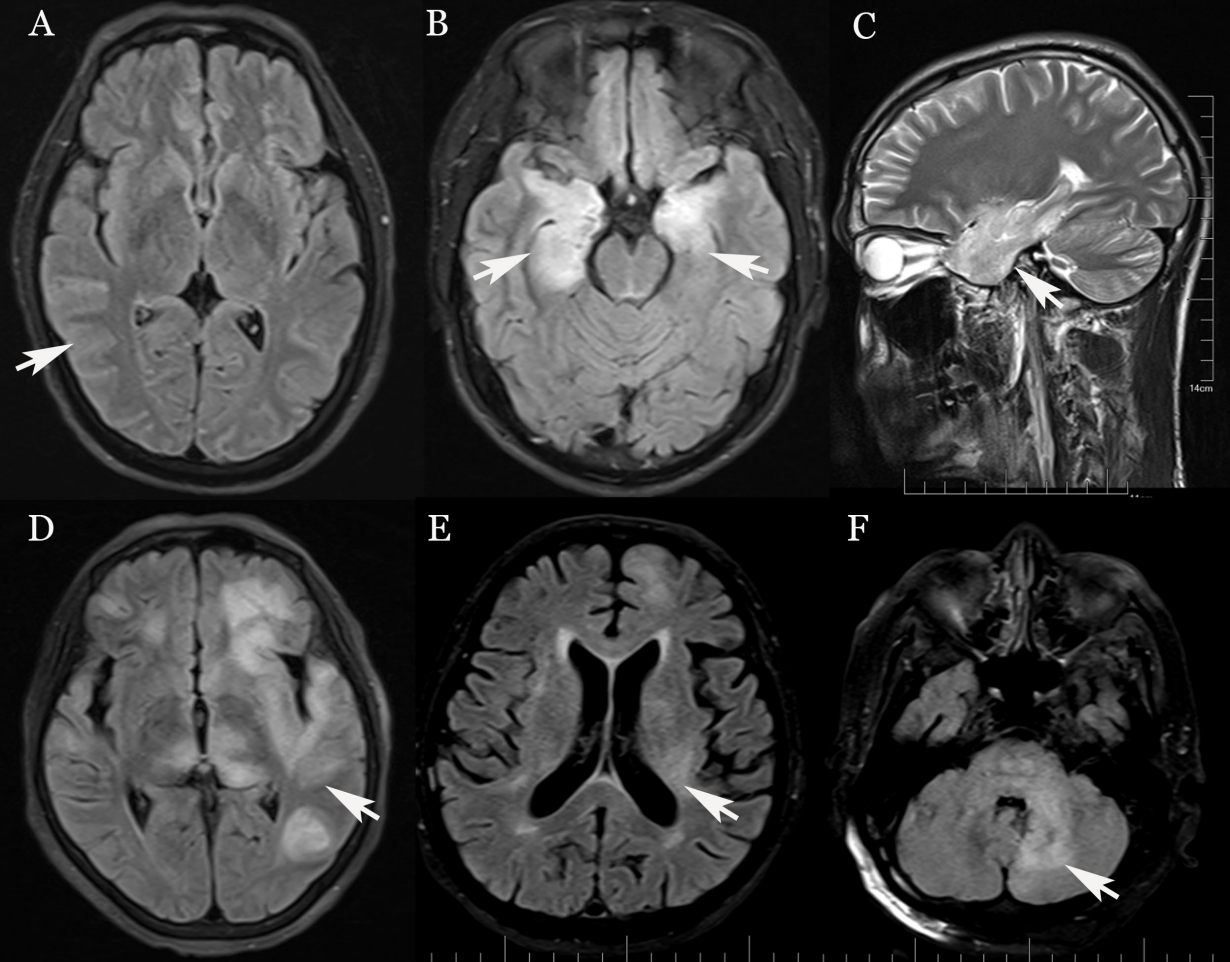
Examples of brain magnetic resonance imaging (MRI) findings of patients. Instructions for brain MRI imaging. All images shown are brain axial fluid-attenuated inversion recovery (FLAIR) images. **(A)** The bilateral hyperintensities in the temporal lobe cortex and insula. Bilateral medial temporal lobe and hippocampal swelling with high signal intensity on FLAIR imaging in **(B, C)** (sagittal sequences). **(D)** Multifocal hyperintensities in both frontal and temporal lobes, basal ganglia region, and left occipital lobe. **(E)** The Left frontal lobe, periventricular abnormal signaling. **(F)** High signal intensity in left cerebellar hemisphere.

**Figure 2:**
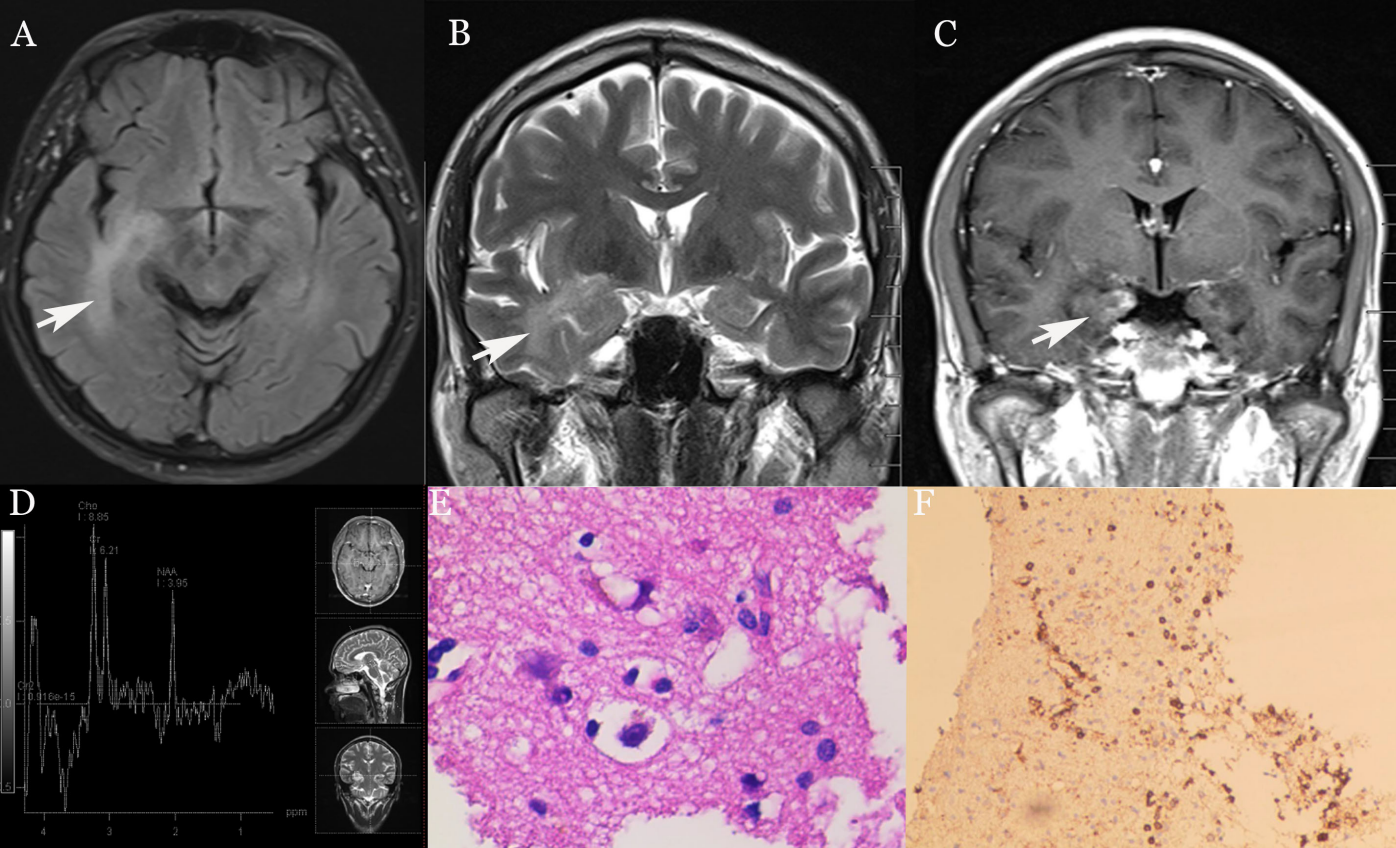
Brain MRI imaging and immunohistochemical analyses of the biopsy. **(A)** Abnormal hyperintensity in the right temporal lobe on brain axial fluid-attenuated inversion recovery (FLAIR) images. The coronal plane **(B)** and post-contrast T1-weighted image **(C)** showed no significant enhancement. **(D)** Magnetic resonance spectroscopy (MRS) revealed a decreased N-acetylaspartate (NAA) peak and an elevated choline (Cho) peak. A biopsy was performed to exclude neoplasm. **(E)** Haematoxylin and eosin (H&E) staining showed perivascular lymphocytic infiltration (original magnification: ×40). **(F)** Immunohistochemical staining revealed that perivascular lymphocytes were positive for CD20 (B cells) and CD3 (T cells). Additionally, reactive glial fibrillary acidic protein (GFAP)-positive astrocytes, scattered synaptophysin-positive cells, and Ki-67-positive inflammatory cells were detected (original magnification: ×10 for immunohistochemical stains).

Low T3 syndrome was observed in 18 patients (23.68%; see **Table 1**). More than half of the patients (77.63%) exhibited abnormal MRI findings (see **Figure 1**). Brain tissue biopsy was performed in one patient to exclude neoplasm. Hematoxylin and eosin (H&E) staining showed perivascular lymphocytic infiltration (see **Figure 2**). Perivascular lymphocytes were CD20+ B cells and CD3+ T cells. Reactive GFAP+ astrocytes, scattered synaptophysin+ cells, and Ki-67+ inflammatory cells were also detected. No cellular atypia was observed. .

Regarding treatment, 69 patients (90.79%) received steroids, 21.05% were administered intravenous immunoglobulin (IVIG), and 16 patients (21.05%) underwent combination therapy. Maintenance therapy was provided to four patients (see **Table 1**).

**Figure 3:**
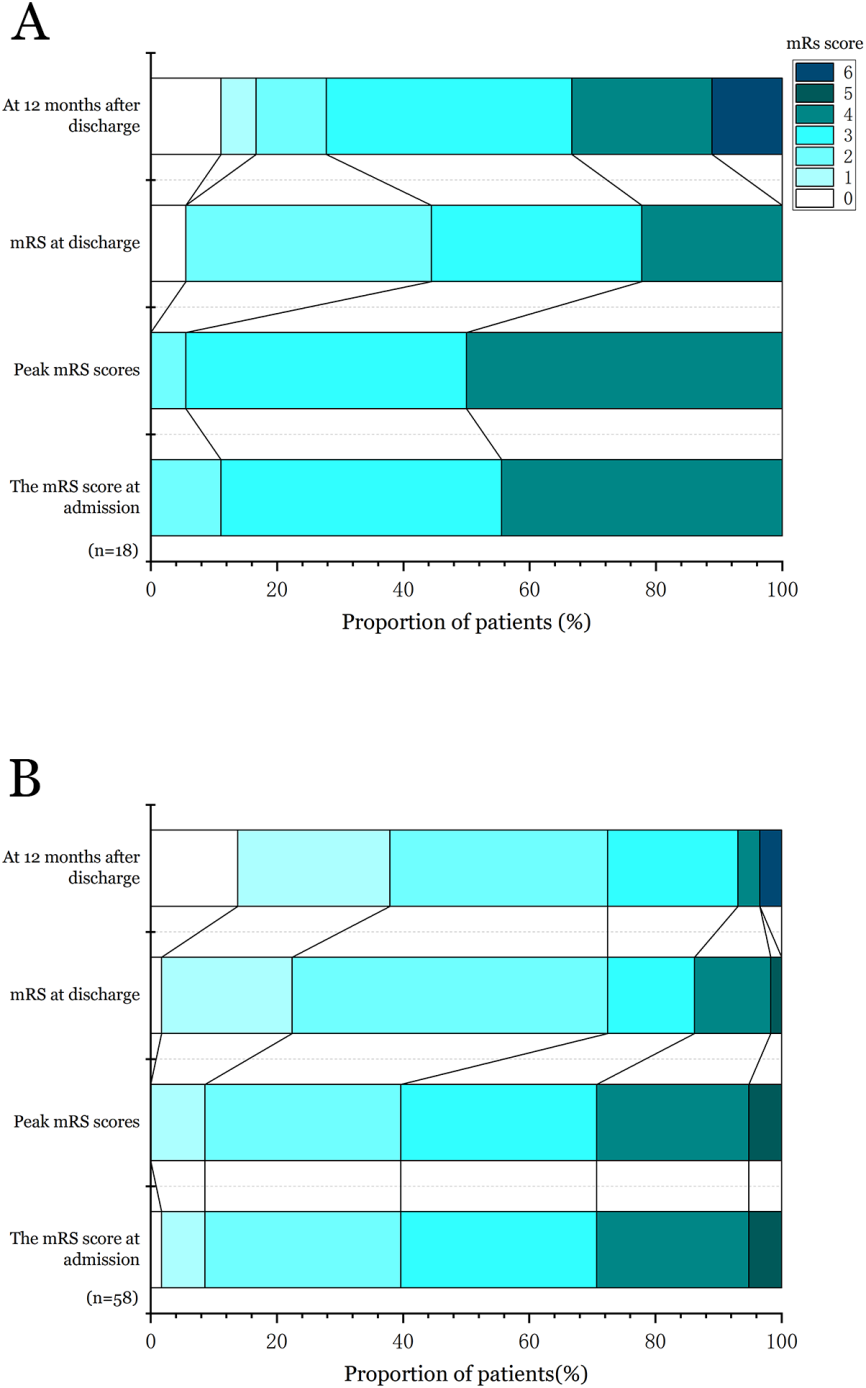
Distribution of mRS scores at admission, peak disease severity, discharge, and at the 12-month discharge follow-up in patients with low T3 syndrome subgroup **(A)** versus those without low T3 syndrome subgroup. **(B)**. mRS, modified Rankin Scale.

All patients were followed up for 12 months. At the end of follow-up, 47 patients (61.84%) had a good prognosis, whereas 29 patients (38.16%) had a poor prognosis (see **Table 1**; **Figure 3**). Four patients died within 12 months after discharge: two from pulmonary infection, one from gastrointestinal bleeding, and one from sepsis.

### Association between fT3 levels and clinical characteristics in patients with antibody-negative AE

As shown in **Table 2**, maximum mRS scores during hospitalization, mRS scores at discharge and at the 12 months follow-up were all significantly and negatively correlated with fT3 levels. In contrast, no significant correlations were found between fT3 levels and the following clinical features: sex, age at onset, length of hospital stay, ICU admission rate, seizures, mental and behavioral disorders, movement disorders, consciousness disorders, urinary and fecal disorders, central hypoventilation, elevated CSF cell count, elevated CSF protein levels, abnormal brain MRI signals, relapse.

**Table 2:** Correlation analysis of factors for low T3 syndrome in patients with antibody-negative autoimmune encephalitis.

Predictive variables	r	P
Sex	-0.046	0.692
Age at onsset	-.o.174	0.133
Hospital stay	-0.005	0.964
ICU admission	-0.117	0.313 0.758
Fever	-0.036	
Headache	0.2	0.083
Psychiatric symptoms	-0.097	0.405
Seizure	0.012	0.919
RSE	-0.087	0.453
Movement disorders	-0.204	0.077
Autonomic dysfunction	-0.108	0.354
Altered consciousness	-0.221	0.055
Bladder dysfunction	-0.105	0.366
Central hypoventilation	-0.146	0.208
Abnormal MRI findings	-0.175	0.131
Elevated white cell count in CSF(>5/μL)	0.148	0.202
Elevated protein in CSF(>45 mg/dL)	0.123	0.29
The mRS score at admission	-0.256	0.026 *
Peak mRS scores	-0.299	0.009 *
mRS at discharge	-0.255	0.026 *
mRS at 12 months after discharge	-0.237	0.039 *
Relapse	-0.041	0.725

ICU, intense care unit; RSE, Refractory status epilepticus; MRI,magnetic resonance imaging; CSF, cerebrospinal fluid; mRS, modified Rankin scale. *P<0.05.

### Correlation between the fT3 and 12 months outcome

In univariable analysis, low fT3 was associated with poor prognosis. Additionally, age at onset, female sex, baseline mRS, hospital stay, hypertension, elevated protein, hippocampus, infratentorial involvement related to poor prognosis (see **Supplementary Table 1**). In subsequent multivariate logistic regression analyses, low fT3 was not significantly associated with poor outcome (OR 0.870, 95% CI 0.592-1.278, p = 0.478). In contrast, discharge mRS score remained significantly associated with poor outcome (OR 0.293, 95% CI 0.103-0.834, p = 0.021; see **Table 3**).

**Table 3:** Multivariate logistic regression analysis of fT3 and discharge mRS score for 12 months unfavorable clinical prognosis.

Variables	OR	95% CI	p value
fT3	0.870	0.592-1.278	0.478
mRS at discharge	0.293	0.103-0.834	0.021

OR, odd ratio; CI, confidence interval; fT3, free triiodothyronine; mRS, modified Rankin scale;.

*P < 0.05.

### Analysis of diagnostic reclassification over the follow-up period

Eight patients (reclassified group) who were initially diagnosed with autoimmune encephalitis were reclassified during disease progression and follow-up. Among them, three were subsequently diagnosed with paraneoplastic syndromes, one was diagnosed with neuropsychiatric systemic lupus erythematosus, and four were diagnosed with non-autoimmune diseases (lymphoma, glioma, and mitochondrial encephalopathy) at the final follow-up (see **Supplementary Table 2**).

Regarding CSF examination at admission, three patients (37.5%) exhibited CSF pleocytosis orelevated total protein, and five patients (62.5%) showed MRI abnormalities (see **Supplementary Figure 1**). Elevated tumor markers (CEA, CA125) were noted in two patients (25%). All patientsreceived immunomodulatory therapy and symptomatic supportive treatment. Among them, three patients (37.5%) experienced adverse reactions related to immunotherapy during the follow-up period after hospitalization or discharge. Additionally, a total of seven patients had a relapse over the course of follow-up, the relapse rate in reclassified group was significantly higher compared to the antibody-negative encephalitis group (p < 0.05) (see **Supplementary Table 3**). Enlargement of pre-existing lesions or development of new lesions on brain MRI was observed in five patients. One case (12.5%) was later diagnosed with lymphoma and died of severe pneumonia that developed as a consequence of inappropriate immunotherapy.

## Discussion

This retrospective study analyzed the clinical features, laboratory profiles, and immunotherapy regimens in a cohort of patients with antibody-negative AE. It specifically investigated the association between low T3 syndrome and both clinical characteristics and long-term prognosis within this population.

We observed that patients with lower fT3 levels within 24 hours of admission experienced a more severe clinical course during hospitalization, including a higher incidence of consciousness disturbances, higher peak mRS scores reached during hospitalization, and significantly higher rates of poor outcomes at the 12-month follow-up. Additionally, a detailed analysis was conducted on the clinical features and immunotherapy-related adverse events in 8 patients who underwent diagnostic revision during follow-up.

In recent years, increasing evidence has indicated that low T3 syndrome is commonly observed in various autoimmune diseases, including multiple sclerosis (MS), NMOSD, Guillain-Barré syndrome, and anti-N-methyl-D-aspartate receptor encephalitis (anti-NMDAR encephalitis) (14–20). A previous study (18) reported that 37.2% of adult patients with anti-NMDAR encephalitis exhibited thyroid dysfunction, among whom 25.6% had low fT3 levels, while another study showed that 15.19% of Chinese patients with anti-NMDAR encephalitis presented with low T3 syndrome (20). In the present study, 23.68% of patients with antibody-negative AE were identified as having low T3 syndrome, consistent with prior reports in autoimmune encephalitis populations. Notably, patients with hyperthyroidism or abnormal thyroid antibodies were excluded from this analysis.

This study also explored the characteristics of patients with antibody-negative AE in combination with low T3 syndrome. Our study found that the low T3 subgroup exhibited a greater incidence of movement disorders and consciousness impairment, as well as a higher maximum mRS score during hospitalization. Among these, mRS score at admission, and peak mRS scores were significantly and negatively associated with low fT3 levels. Previous research has established fT3 as a pivotal regulator of brain development and a modulator of immune system function. Autoimmune diseases may disrupt the homeostatic balance among the central nervous system, endocrine system, and immune system, potentially explaining the observed clinical correlation between low fT3 levels and disease severity (21). However, no significant correlation was found between fT3 levels and the presence of these clinical features. Several factors may explain this discrepancy. First, a potential threshold effect may exist, whereby only severely reduced FT3 levels are associated with an increased frequency of symptoms. Second, the limited sample size may have restricted the power to detect a significant correlation. Larger sample sizes are needed in future studies.

Our study also focused on the predictive performance of low T3 syndrome in the prognosis of patients with antibody-negative AE. Previous studies have demonstrated the prognostic significance of low fT3 in conditions such as autoimmune encephalitis, stroke, and severe brain injury (11, 12, 18, 19). Multivariable analysis revealed that after adjusting for multiple confounders, low fT3 did not retain independent prognostic significance. Prior research suggests that low fT3 levels may reduce neuroprotective effects and exacerbate secondary brain injury following encephalitis (22–24). Moreover, thyroid hormones may also modulate disease severity through their role in neurotrophic factor secretion. We suggest that low fT3 is a non-specific marker of acute illness severity rather than a specific driver of poor outcomes in antibody-negative AE. The relatively small sample size may have limited our statistical power to detect a modest independent effect. Furthermore, residual confounding from unmeasured variables such as nutritional status cannot be ruled out. Notably, discharge mRS emerged as a robust independent predictor, suggesting that functional status at discharge is a clinically meaningful prognostic indicator. Future studies with larger cohorts are needed to clarify the prognostic role of low T3 syndrome.

In our single-center cohort, the misdiagnosis rate of antibody-negative AE was 9.52%, which is comparable to that reported in a retrospective analysis of adult patients with antibody-negative AE from the Mayo Clinic Autoimmune Neurology Clinic (6/51, 11.76%) (25), yet significantly lower than the overall misdiagnosis rate reported in a multicenter retrospective study on autoimmune encephalitis (107/393,27.23%). Functional neurological disorders, neurodegenerative diseases, primary psychiatric conditions, tumors, and infectious diseases are commonly misdiagnosed as AE (26). In our cohort, revised diagnoses were primarily concentrated in tumor-related diseases, systemic autoimmune disorders, and genetic conditions, while misdiagnoses related to functional neurological disorders, primary psychiatric illnesses, or infections were less frequent. These findings highlight the importance of a comprehensive clinical assessment, including detailed medical history and close follow-up, in the diagnosis of antibody-negative AE. Notably, the clinical features and laboratory tests was similar in the two groups, and the recurrence rate of reclassified group was higher than antibody-negative AE group. Notably, the clinical features and laboratory tests was similar in the two groups, however, the recurrence rate of reclassified group was higher than antibody-negative AE group, with more pronounced clinical and imaging-based relapses. Based on our case review, we propose several warning signs that should prompt reconsideration of the initial antibody-negative AE diagnosis: (1) a relapsing or fluctuating disease course rather than a monophasic illness; (2) only transient or incomplete response to first-line immunotherapy (corticosteroids or intravenous immunoglobulin); (3) progressive abnormalities on brain MRI that do not follow typical antibody-negative AE patterns; (4) abnormal tumor markers. When such red flags are present, clinicians should maintain a broad differential diagnosis including tumors, infections, metabolic disorders, genetic diseases, and systemic autoimmune conditions. In selected cases, brain biopsy may be necessary to establish a definitive diagnosis.

Nevertheless, several limitations of this study should be acknowledged. First, owing to its single-center retrospective design, the study is susceptible to selection and recall biases. Second, the retrospective nature precluded a detailed assessment of treatment responses, including time to response and recovery patterns of specific neurological deficits, which restricts the use of more precise assessment methods. Third, mild CSF abnormalities may reduce diagnostic specificity, thereby leading to an increased risk of misdiagnosis. Fourth, the small sample size of the low T3 subgroup limits the robustness of our conclusions. Accordingly, larger-scale, multicenter prospective studies are warranted to validate and extend our findings.

## Conclusion

This study investigated low T3 syndrome in patients with antibody-negative AE, a condition with limited existing literature. Our findings showed that low T3 may reflect general disease severity rather than serving as an independent prognostic biomarker. Discharge mRS appears to be a more robust independent predictor. Additionally, analysis of reclassified patients revealed nonspecific clinical and laboratory manifestations, reflecting a diagnostic challenge intrinsic to antibody-negative AE. Relapsing course, incomplete immunotherapy response, progressive atypical MRI findings, and elevated non-paraneoplastic tumor markers are red flags that warrant re-evaluation of antibody-negative AE diagnosis. Larger prospective studies are needed.

## Data Availability

The original contributions presented in the study are included in the article/**Supplementary Material**, further inquiries can be directed to the corresponding author/s.

## Supplementary material

Supplementary Figure 1 Brain MRI imaging findings from patients with revised diagnoses during follow-up. Brain axial fluid-attenuated inversion recovery (FLAIR) images from patient No. 2 showed an abnormal hyperintensity within the hippocampal region **(A)**. Follow-up MRI revealed significant interval progression of a right temporal lobe lesion, characterized by marked enlargement and substantial perilesional edema **(B, C)**. Histopathological confirmation was subsequently obtained via biopsy, establishing the definitive diagnosis of glioma. Brain axial FLAIR image from patient No. 4 demonstrated multiple abnormal signal foci involving the right parietal lobe, bilateral inferior frontal regions, and the left temporal lobe **(D, E)**. Post-contrast imaging **(F)** revealed prominent gyriform enhancement in the left temporal lobe. Finally, genetic analysis suggested a mitochondrial encephalopathy.
