# Supplementary figures and images for: Clinical implications of free triiodothyronine levels and diagnostic revisions in antibody-negative autoimmune encephalitis
Source: Front Immunol. 2026 Jul 7;17:1847846. doi: 10.3389/fimmu.2026.1847846 (PMC13384918; doi:10.3389/fimmu.2026.1847846)

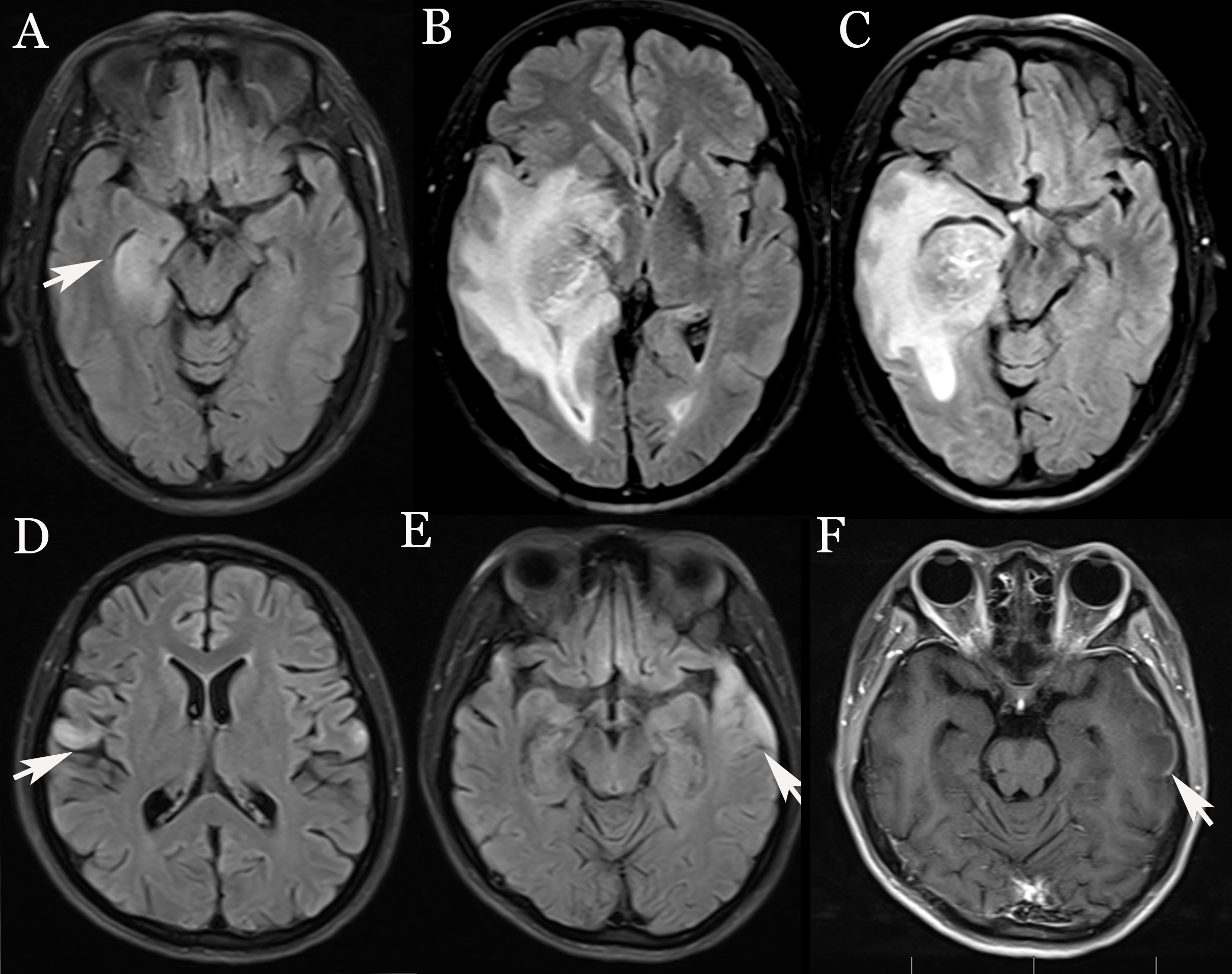

Supplement: Supplementary Figure 1 — Brain MRI imaging findings from patients with revised diagnoses during follow-up. Brain axial fluid-attenuated inversion recovery (FLAIR) images from patient No. 2 showed an abnormal hyperintensity within the hippocampal region (A). Follow-up MRI revealed significant interval progression of a right temporal lobe lesion, characterized by marked enlargement and substantial perilesional edema (B, C). Histopathological confirmation was subsequently obtained via biopsy, establishing the definitive diagnosis of glioma. Brain axial FLAIR image from patient No. 4 demonstrated multiple abnormal signal foci involving the right parietal lobe, bilateral inferior frontal regions, and the left temporal lobe (D, E). Post-contrast imaging (F) revealed prominent gyriform enhancement in the left temporal lobe. Finally, genetic analysis suggested a mitochondrial encephalopathy. [file Image1.tif]
