# Supplementary material for: Clinical implications of free triiodothyronine levels and diagnostic revisions in antibody-negative autoimmune encephalitis
Source: Front Immunol. 2026 Jul 7;17:1847846. doi: 10.3389/fimmu.2026.1847846 (PMC13384918; doi:10.3389/fimmu.2026.1847846)
Supplement: Supplementary file 2 [file SupplementaryFile1.docx]

**Supplemental Table 1. Demographics and clinical features based on 12 months outcome.**

|  | Total  (n =76) | Good-prognosis  Subgroup  (n =47 ) | Poor-prognosis  subgroup  (n =29) | P value |
| --- | --- | --- | --- | --- |
| **Demographics** |  |  |  |  |
| Age at onset, (median, IQR) | 44（14-85） | 40(15-81) | 55(14-85) | 0.026 * |
| Age of onset ≥60 | 18(23.68) | 9 (19.15) | 9(31.03) | 0.236 |
| Female sex ,n(%) | 22(28.95) | 17(36.17) | 5（17.24） | 0.077 * |
| **Clinical profiles** |  |  |  |  |
| The mRS score at admission, mean (SD) | 2.96±1.03 | 2.62±0.99 | 3.52±0.83 | ＜0.001 * |
| Peak mRS scores, mean (SD) | 3.0±0.99 | 2.66±0.94 | 3.55±0.83 | ＜0.001 * |
| mRS at discharge, mean (SD) | 2.30±1.03 | 1.89±0.84 | 2.97±0.98 | ＜0.001 * |
| Hospital stay, median (IQR), days | 13(3-84) | 10(3-56) | 16(3-84) | 0.031 * |
| **ICU admission**,n(%) | 5(6.58) | 2(4.26) | 3(10.35) | 0.573 |
| **Comorbidities,** n (%) |  |  |  |  |
| Hypertension | 13(17.11) | 5(10.64) | 8(27.59) | 0.057 * |
| Diabetes | 8(10.53) | 5(10.64) | 3(10.35) | 1 |
| Tumor | 2(2.63) | 0 | 2(3.45) | 1 |
| Elevated tumor markers | 3(3.95) | 3(6.38) | 0 | 0.434 |
| Other concomitant  non-neural autoantibodies in the serum | 9(11.84) | 2(4.26) | 7(12.07) | 1 |
| **Prodromal symptoms** | 10(13.16) | 7(14.89) | 3(10.35) | 0.825 |
| **Clinical syndrome,**n(%) |  |  |  |  |
| Fever | 22(28.95) | 13(26.66) | 9(31.03) | 0.797 |
| Headache | 26(34.21) | 18(38.30) | 8(27.59) | 0.339 |
| Psychiatric symptoms | 18(23.68) | 10(21.28) | 8(27.59) | 0.530 |
| Seizure | 35(46.05) | 24(51.06) | 11(37.93) | 0.265 |
| RSE | 8(10.53) | 3(6.38) | 5(17.24) | 0.265 |
| Movement disorders | 20(26.32) | 10(21.28) | 10(34.48) | 0.204 |
| Autonomic dysfunction | 6(7.89) | 3(6.38) | 3(10.35) | 0.854 |
| Altered consciousness | 9(11.84) | 4(8.51) | 5(17.24) | 0.252 |
| Bladder dysfunction | 4(5.26) | 3(6.38) | 1(3.45) | 0.978 |
| Central hypoventilation | 6(7.89) | 2(4.26) | 4(13.79) | 0.289 |
| **Blood tests** |  |  |  |  |
| WBC count, median (IQR),  n× 10^9^ /L | 8.34（2.79-17.1） | 8.28（4.33-16.14） | 8.40（2.79-17.1） | 0.991 |
| CRP, median (IQR), mg/L | 3.41（0.5-132） | 2.99（0.5-136.25） | 3.76（0.5-161） | 0.646 |
| D-Dimer，median (IQR), ug/L | 465（50-4950） | 260（158-489） | 250（88-381） | 0.229 |
| **Thyroid status** |  |  |  |  |
| fT3, median (IQR), pmol/L | 4.42（0.92-11.91） | 4.50(1.18-11.91) | 3.9（0.92-12.02） | 0.002* |
| fT4, median (IQR), pmol/L | 11.6（7.39-17.76） | 11.41（7.39-18.43） | 12.1（8.25-17.76） | 0.82 |
| TSH, median (IQR), uIU/ml | 1.15（0.11-4.57） | 1.17（0.11-3.38） | 1.09（0.22-4.76） | 0.97 |
| **CSF findings,** n(%) |  |  |  |  |
| Increased intracranial pressure (cmH2O) | 18(23.68) | 12(25.53) | 6(20.69) | 0.63 |
| Elevated white cell count (>5/μL) | 23(30.26) | 15(51.72) | 8(27.59) | 0.69 |
| Elevated white cell count  (> 20 white cell count/μl) | 18(23.68) | 10(21.28) | 8(27.59) | 0.53 |
| Elevated protein (>45 mg/dL) | 37(48.68) | 19(40.43) | 18（62.07） | 0.067 * |
| **MRI finding,** n(%) |  |  |  |  |
| Abnormal T2WI/FLAIR hyperintensities | 59(77.63) | 37(78.72) | 22（75.86） | 0.771 |
| Cortex | 32(42.11) | 17(36.17) | 15（51.72） | 0.182 |
| White matter | 14(18.42) | 8(17.02) | 6（20.69） | 0.689 |
| Basal ganglia | 11(14.47) | 6(12.77) | 5(17.24) | 0.59 |
| Thalamus | 6(7.89) | 3(6.38) | 3(10.35) | 0.534 |
| Corpus callosum | 5(6.58) | 2(4.26) | 3(10.35) | 0.573 |
| Hippocampus | 21(27.63) | 17(36.17) | 4(13.79) | 0.064 * |
| Brainstem | 10(13.16) | 4(8.51) | 6(20.69) | 0.239 |
| Cerebellum | 10(13.16) | 5(10.64) | 5(17.24) | 0.408 |
| infratentorial involvement  **EEG abnormalities,**n(%) | 16(21.05)  24(31.58) | 6(12.77)  8(17.02) | 10(34.48)  16（55.17） | 0.024 *  0.556 |
| **Treatment**, n(%) |  |  |  |  |
| First-line immunotherapy |  |  |  |  |
| Steroids | 69(90.79) | 42(89.36) | 27（93.10） | 0.889 |
| IVIg | 16(21.05) | 8(17.02) | 8(27.59) | 0.272 |
| Combined immunotherapy | 16(21.05) | 8(17.02) | 8(27.59) | 0.391 |
| Second-line immunotherapy |  |  |  |  |
| Delay of immunotherapy for ≥1 month | 23(30.26) | 16(34.04) | 7（24.14） | 0.361 |
| Long-course immunotherapy | 4(5.26) | 3(6.38) | 1（3.45） | 0.978 |
| **Follow‐up outcomes**, n(%) |  |  |  |  |
| mRS at 12 months after discharge, mean (SD) | 2.16±1.46 | 1.26±0.79 | 3.62±1.05 | ＜0.001 * |
| Relapse | 26(38.16) | 15(51.72) | 11（37.93） | 0.591 |

**Note:** IQR, interquartile range; SD, standard deviation; RSE, Refractory status epilepticus; MRI,magnetic resonance imaging; WBC, white blood cell; CRP, c-reactive protein; CSF, cerebrospinal fluid; fT3, free triiodothyronine; fT4, free thyroxine; TSH, thyroid stimulating hormone; ICU, intense care unit; mRS, modified Rankin scale;IVIg, intravenous immunoglobulin. *P<0.1.
