# Supplementary material for: Clinical implications of free triiodothyronine levels and diagnostic revisions in antibody-negative autoimmune encephalitis
Source: Front Immunol. 2026 Jul 7;17:1847846. doi: 10.3389/fimmu.2026.1847846 (PMC13384918; doi:10.3389/fimmu.2026.1847846)
Supplement: Supplementary file 3 [file SupplementaryFile2.docx]

**Supplemental Table 2. Patients with revised diagnoses over the follow-up course.**

| No. | Age/  sex | Final diagnosis | Clinical  onset | Brief description | Immunotherapy-related adverse events |
| --- | --- | --- | --- | --- | --- |
| 1 | F/70 | Paraneoplastic encephalitis | Acute | Hallucinations and confusion,  Cerebrospinal fluid analysis demonstrated elevated protein and pleocytosis, and ‌carcinoma of the rectum was detected during follow-up‌. | None |
| 2 | M/58 | Glioma | Subacute | A patient with autonomic dysfunction initially improved on steroids and IVIG, but later deteriorated with radiographic progression of a hippocampal lesion. Biopsy ultimately confirmed glioma. | Alimentary tract  hemorrhage |
| 3 | F/38 | Primary central nervous system diffuse large B-cell lymphoma | Subacute | The patient, initially admitted for limb numbness and weakness, relapsed six months after steroid pulse therapy. Cranial MRI showed markedly progressive lesions, leading to a final diagnosis of primary central nervous system diffuse large B-cell lymphoma. | None |
| 4 | F/23 | Mitochondrial  encephalopathy | Subacute | A patient presenting with behavioral changes and seizures had steroid-responsive MRI abnormalities. Following relapse, a positive lactate stress test and genetic analysis led to the diagnosis of mitochondrial encephalopathy. | None |
| 5 | F/57 | Paraneoplastic encephalitis | Chronic | A patient whose initial MRI and CSF findings were normal later developed a right temporal lobe lesion and was found to have endometrial carcinoma. | Urinary system  infection |
| 6 | M/53 | [Primary central](https://pubmed.ncbi.nlm.nih.gov/31187523/)  [nervous system lymphoma](https://pubmed.ncbi.nlm.nih.gov/31187523/) | Chronic | A patient with hypoventilation, movement disorders, and multifocal brain lesions responded initially to steroids and IVIG but relapsed.Biopsy ultimately confirmed [primary central](https://pubmed.ncbi.nlm.nih.gov/31187523/)  [nervous system lymphoma](https://pubmed.ncbi.nlm.nih.gov/31187523/). | Severe pneumonia |
| 7 | F/28 | Neuropsychiatric systemic lupus erythematosus | Acute | A patient who initially presented with mildly elevated ANA levels and normal complement levels subsequently developed thrombocytopenia, and follow-up testing confirmed the presence of positive anti-dsDNA antibodies. | None |
| 8 | M/57 | Paraneoplastic encephalitis | Subacute | A patient with an initially normal brain MRI and subsequent diagnosis of malignant lung tumor had an EEG showing abnormal temporal discharges. | Severe pneumonia |

**Note:** MRI, magnetic resonance imaging; IVIG ,intravenous immunogloblin; ANA, Antinuclear antibody ; anti-dsDNA，anti-double-stranded DNA, EEG,encephalogram; IVIg, intravenous immunoglobulin; CSF, cerebrospinal fluid;
