# Supplementary material for: Clinical implications of free triiodothyronine levels and diagnostic revisions in antibody-negative autoimmune encephalitis
Source: Front Immunol. 2026 Jul 7;17:1847846. doi: 10.3389/fimmu.2026.1847846 (PMC13384918; doi:10.3389/fimmu.2026.1847846)
Supplement: Supplementary file 4 [file SupplementaryFile3.docx]

**Supplemental Table 3. Clinical data of patients with antibody-negative autoimmune encephalitis and reclassified patients.**

|  | Reclassified group  (n = 8) | Antibody-negative AE group  (n = 76) | p value |
| --- | --- | --- | --- |
| **Demographics** |  |  |  |
| Age at onset, (median, IQR) | 55（23-70） | 44（14-85） | 0.796 |
| Age of onset ≥60,n(%) | 1(12.50) | 18(23.68) | 0.783 |
| Female sex ,n(%) | 5（62.50） | 22(28.95) | 0.125 |
| **Clinical profiles** |  |  |  |
| The mRS score at admission, mean (SD) | 3.25±1.16 | 2.96±1.03 | 0.455 |
| Peak mRS scores, mean (SD) | 3.38±1.06 | 3.0±0.99 | 0.316 |
| mRS at discharge, mean (SD) | 2.50±1.41 | 2.30±1.03 | 0.621 |
| Hospital stay, median (IQR), days | 12(6-33) | 13(3-84) | 0.737 |
| **ICU admission**,n(%) | 1(12.50) | 5(6.58) | 1 |
| **Comorbidities,** n (%) |  |  |  |
| Hypertension | 0 | 13(17.11) | 0.448 |
| Diabetes | 0 | 8(10.53) | 0.740 |
| Elevated tumor markers | 2(25.00) | 3(3.95) | 0.108 |
| Other concomitant  non-neural autoantibodies in the serum | 3(37.50) | 9(11.84) | 0.149 |
| Prodromal symptoms | 2(25.00) | 10(13.16) | 0.704 |
| **Clinical syndrome,**n(%) |  |  |  |
| Fever | 3(37.50) | 22(28.95) | 0.942 |
| Headache | 0 | 26(34.21) | 0.112 |
| Psychiatric symptoms | 3(37.50) | 18(23.68) | 0.668 |
| Seizure | 2(25.00) | 35(46.05) | 0.443 |
| RSE | 0 | 8(10.53) | 0.740 |
| Movement disorders | 3(37.50) | 20(26.32) | 0.796 |
| Autonomic dysfunction | 1(12.50) | 6(7.89) | 1 |
| Altered consciousness | 2(25.00) | 9(11.84) | 0.618 |
| Bladder dysfunction | 1(12.50) | 4(5.26) | 0.978 |
| Central hypoventilation | 2(25.00) | 6(7.89) | 0.350 |
| **Blood tests** |  |  |  |
| WBC count, median (IQR), n× 109 /L | 8.86（4.1-13.93） | 8.34（2.79-17.1） | 0.670 |
| CRP, median (IQR), mg/L | 5.69（0.5-15.02） | 3.41（0.5-132） | 0.994 |
| D-Dimer,median (IQR), ug/L | 435（120-3600） | 465（50-4950） | 0.988 |
| **Thyroid status** |  |  |  |
| fT3, median (IQR), pmol/L | 4.08（2.37-7.54） | 4.42（0.92-11.91） | 0.743 |
| fT4, median (IQR), pmol/L | 11.79（7.99-19.43） | 11.6（7.39-17.76） | 0.873 |
| TSH, median (IQR), uIU/ml | 1.08（0.12-2.12） | 1.15（0.11-4.57） | 0.563 |
| **CSF findings,**n(%) |  |  |  |
| Increased intracranial pressure (cmH2O) | 2(25.00) | 18(23.68) | 1 |
| Elevated white cell count (>5/μL) | 3(37.50) | 23(30.26) | 0.985 |
| Elevated white cell count  (> 20 white cell count/μl) | 2(25.00) | 18(23.68) | 0.934 |
| Elevated protein (>45 mg/dL) | 3(37.50) | 37(48.68) | 0.818 |
| Decreased glucose level (< 2.5 mmol/L) | 1(12.50) | 12(15.79) | 1 |
| **MRI finding,**n(%) |  |  |  |
| Abnormal T2WI/FLAIR hyperintensities | 5(62.50) | 59(77.63) | 0.603 |
| Infratentorial involvement | 2(25.00) | 16(21.05) | 1 |
| **EEG abnormalities,**n(%) | 5(62.50) | 24(31.58) | 0.174 |
| **Prognosis** |  |  |  |
| Poor outcome (mRS 3–6), n (%) | 3(37.50) | 29(38.16) | 1 |
| mRS scores at 12 months after discharge | 2.75±2.05 | 2.16±1.46 | 0.298 |
| Relapse,n(%) | 7(87.50) | 26(38.16) | 0.011 * |
| Death,n(%) | 1(12.50) | 4(5.26) | 0.970 |

**Note:** AE, autoimmune encephalitis; IQR, interquartile range; SD, standard deviation; RSE, Refractory status epilepticus; fT3, free triiodothyronine; fT4, free thyroxine; TSH, thyroid stimulating hormone. MRI, magnetic resonance imaging; WBC, white blood cell; CRP, c-reactive protein; CSF, cerebrospinal fluid; ICU, intense care unit; EEG,encephalogram; mRS, modified Rankin scale. *P<0.05.
